# Wealth Inequality and Tuberculosis Elimination in Europe

**DOI:** 10.3201/eid1511.090916

**Published:** 2009-11

**Authors:** Jonathan E. Suk, Davide Manissero, Guido Büscher, Jan C. Semenza

**Affiliations:** European Centre for Disease Prevention and Control Scientific Advice Unit, Stockholm, Sweden (J.E. Suk, D. Manissero, G. Büscher, J.C. Semenza); University of Cologne Institute of Health Economics and Clinical Epidemiology, Cologne, Germany (G. Büscher)

**Keywords:** Tuberculosis and other mycobacteria, socioeconomic factors, Europe, European Union, financial crisis, bacteria, dispatch

## Abstract

In Europe, wealth inequality is directly related to tuberculosis (TB) notification (R^2^ = 0.69), while in countries with lower TB rates, higher proportions of TB cases occur in foreign-born persons. Particularly during times of financial upheaval, efforts to eliminate TB must address social inequality.

The current global financial crisis may be expected to exacerbate health inequalities (*1*), which in turn lead to differential health outcomes (*2**,**3*). In Europe, for example, discrepancies between those living in lower and higher socioeconomic positions are manifested through differential death rates from chronic diseases, such as cardiovascular and cerebrovascular diseases, as well as alcohol- and smoking-related diseases (*4*).

Similar discrepancies also exist for communicable diseases. A comprehensive literature review demonstrated that in every European Union (EU) member state, vulnerable groups (those with low educational or income levels, migrants, persons engaged in high-risk lifestyles) have a disproportionately higher incidence of communicable diseases (*5*). However, because the overall effect of communicable diseases is currently estimated to be 9% of total diseases in Europe, such differences are difficult to quantify (*6*). Furthermore, surveillance systems do not systematically capture indicators of socioeconomic status (such as education, occupation, ethnicity, or housing tenure) or link those indicators to specific persons.

Tuberculosis (TB) provides a good case study for further analyzing correlations between communicable diseases and wealth distribution. Historically, the decline of TB incidence in Europe preceded the advent of anti-TB drugs and coincided with rapid improvement of quality of life (*7*). Whether this link continues to be valid for high-income countries remains an open question. Earlier studies carried out after the resurgence of TB in the late 1980s in North America and in Europe indicated that, along with HIV infection and drug resistance, socioeconomic factors were a major determinant in acquiring TB (*8**,**9*). The 27 EU member states, with a wide distribution of TB notification rates (4–138/100,000 population/year) as well as diverse levels of wealth as measured by gross domestic product (GDP) in purchasing power standards (PPS) per capita (8,600–63,100) (Eurostat; http://epp.eurostat.ec.europa.eu), represent an optimal setting in which to analyze whether a correlation can be detected between wealth, social cohesion, and TB.

## The Study

In this ecologic study, distribution of TB prevalence rates (all forms, per 100,000 population per year, 2006) (*10*) for each EU member state was plotted against 2 measures of income distribution to examine the most descriptive indicator of how socioeconomic setting relates to TB prevalence in Europe: 1) the Gini coefficient, a common measure of inequality of income distribution within a country (*11*); and 2) Eurostat’s inequality of income distribution ratio, which measures the ratio of total income received by the 20% of the population with the highest income (top quintile) to that received by the 20% of the population with the lowest income (lowest quintile). The Gini coefficient was not strongly associated with TB prevalence in Europe (R^2^ = 0.22), nor was Eurostat’s inequality of income distribution ratio (R^2^ = 0.34).

Hypothesizing that the quantification of a country’s wealth (i.e., GDP), along with its distribution, would correlate better than either indicator separately, we computed an indicator called the public wealth index (PWI). This index divides a nation’s economic wealth (using Eurostat data on GDP in PPS per capita) by its level of social cohesion (using the Eurostat inequality of income distribution ratio). Effectively, this metric takes the relative high level of wealth in Europe into account while also controlling for its distribution. It favors wealthy countries with low ratios of income inequality: the top 5 scores on the public wealth index were generated by Luxembourg, Norway, Denmark, Sweden, and the Netherlands.

Using the PWI, we then developed a simple regression model to explain TB prevalence rates. Because of the structure of the data, we used a log-log transformation in R version 2.8 (*12*). The explanatory variable (PWI) and the dependent variable (TB prevalence rates), were log-transformed. We analyzed all 27 EU member states as well as Norway and Iceland. The model yielded a strong inverse relationship between PWI and TB rates with a correlation coefficient of R^2^ = 0.69. The differences when using the estimator for the intercept parameter in the model (14.36, p<0.001) and when using the estimated regression parameter for the logarithmic PWI (–1.39, p<0.001) were both significant. The observed values and the regression line with no log transformation are shown in the Figure.

**Figure:**
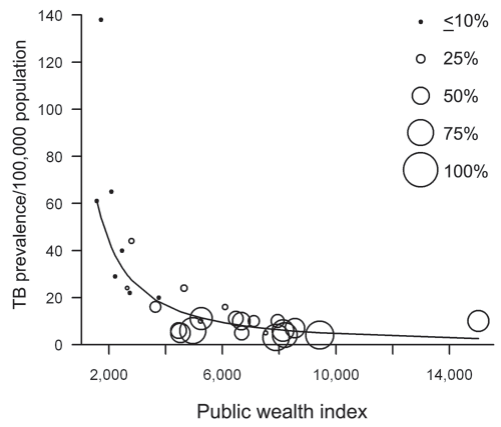
Public wealth index and tuberculosis (TB) prevalence rates in the 27 European Union member states plus Norway and Iceland, 2006. GDP, gross domestic product.

Finally, to demonstrate the differences in the composition of TB populations between countries, we plotted the percentage of foreign-born TB case-patients within a country (a surveillance proxy for immigrant populations, which is typically defined as place of birth, except in Austria, Belgium, Bulgaria, Malta, and Poland, where it is defined as place of citizenship as reported in 2006) (*10*). As countries rank higher on the PWI, the proportion of TB case-patients that are foreign-born generally increased (Figure). With increasing PWI status, TB rates dropped, but the proportion of foreign-born TB case-patients increased.

## Conclusions

We demonstrate a strong inverse relationship between PWI scores and TB rates. The data presented here are, however, subject to important limitations. First, aggregation bias is inherent in all ecologic studies, which are not able to disaggregate individual level risk factors important in TB transmission. Second, national-level surveillance information consists of few socioeconomic indicators. One of the indicators, foreign born, is perhaps an unfortunate proxy term for migrant populations, but it is the only one available. The term is further limiting because definitions of foreign born vary between countries, as discussed earlier. Third, inconsistencies in TB reporting likely occur across the European Union, although this would in any case bias the results away from the null hypothesis.

Nevertheless, given the strong correlation between the PWI and TB rates across Europe, as well as the strong trend linking high PWI with higher rates of TB among foreign-born populations, our data lend support to the notion of ensuring equality, both within and between nations, as an important building block for effective TB control. Yet, as the Figure suggests, especially for countries with higher scores on the PWI, emphasis must also be placed on directly engaging specific vulnerable groups for public health action, whether these groups consist of foreign-born persons, HIV-positive persons, Roma people (http://web.worldbank.org/WBSITE/EXTERNAL/COUNTRIES/ECAEXT/EXTROMA/0,,contentMDK:20341647~menuPK:648308~pagePK:64168445~piPK:64168309~theSitePK:615987,00.html), or others.

The current financial crisis could exacerbate the conditions of existing vulnerable groups as well as create new ones. For example, the EU Directorate for Employment, Social Affairs and Equal Opportunities estimates that 16% of Europe’s population currently lives below the poverty line (*13*). Rising unemployment rates could push this rate even higher, with implications for factors that drive TB spread such as the quality of housing and sanitation. Returning jobless migrants might also be particularly vulnerable if they are no longer able to access their country’s social insurance systems. Thus, particularly for countries with high incidences of TB, advancing social equalities is fully compatible with the aim of lowering TB prevalence rates. Indeed, addressing social and environmental determinants (such as social inclusion, education and training, crowding, and indoor air pollution) could pay dividends in the fight against TB during difficult economic times.

Data related to the financial crisis and its effects on public health will need to be carefully scrutinized as they become available. In the meantime, the public health community must continue to both defend and act upon the insights from the World Health Organization Commission on the Social Determinants of Health, which has so eloquently inserted discussion of social inequalities into public health discourse (*3*). Addressing TB among vulnerable populations and tailoring services to these groups (*14*) will also be an essential component of any strategy aiming at progressing towards TB elimination, as the action plan by the European Centre for Disease Prevention and Control suggests (*15*).
